# Effects of Succinate on Growth Performance, Meat Quality and Lipid Synthesis in Bama Miniature Pigs

**DOI:** 10.3390/ani14070999

**Published:** 2024-03-25

**Authors:** Xiangyun Zhai, Liping Dang, Shiyu Wang, Wenyuan Li, Chao Sun

**Affiliations:** 1 College of Animal Science and Technology, Northwest A&F University, Yangling 712100, China; zhaixiangyun2016@126.com (X.Z.); dangliping2017@163.com (L.D.); 13607658576@163.com (S.W.); 2 Agriculture and Rural Bureau of Yuanyang County, Xinxiang 453000, China; liwenyuan29@126.com

**Keywords:** Bama miniature pigs, succinate, lipid deposition, meat quality

## Abstract

**Simple Summary:**

Succinate is an important product of the fermentative metabolism of dietary fiber, and similar to propionate, high levels of succinate in the gut modulate the body’s inflammatory response, glucose tolerance, and lipid metabolism. Our study showed that the addition of 1% succinate to pig diets promoted growth and fat deposition capacity without affecting feed intake, remodeled the fatty acid content of the longissimus dorsi muscle, and consequently improved meat quality. Further, we isolated and cultured primary porcine preadipocytes. The results showed that succinate increased the expression of genes related to adipogenic differentiation while decreasing the levels of genes related to lipolysis and promoting the formation of cellular lipid droplets. Mechanistically, dietary supplementation with succinate increases succinylation modification in adipose tissue to promote facilitated lipogenesis. This suggests that it is feasible and important to improve fatty acids and meat quality in porcine muscle through dietary succinate supplementation.

**Abstract:**

Succinate, one of the intermediates of the tricarboxylic acid cycle, is now recognized to play a role in a broad range of physiological and pathophysiological settings, but its role in adipogenesis is unclear. Our study used Bama miniature pigs as a model to explore the effects of succinate on performance, meat quality, and fat formation. The results showed that adding 1% succinate significantly increased the average daily gain, feed/gain ratio, eye muscle area, and body fat content (*p* < 0.05), but had no effect on feed intake. Further meat quality analysis showed that succinate increased the marbling score and intramuscular fat content of longissimus dorsi muscle (LM), while decreasing the shear force and the cross-sectional area of LM (*p* < 0.05). Metabolomics analysis of LM revealed that succinate reshaped levels of fatty acids, triglycerides, glycerophospholipids, and sphingolipids in LM. Succinate promotes adipogenic differentiation in porcine primary preadipocytes. Finally, dietary succinate supplementation increased succinylation modification rather than acetylation modification in the adipose tissue pool. This study elucidated the effects of succinate on the growth and meat quality of pigs and its mechanism of action and provided a reference for the role of succinate in the nutrition and metabolism of pigs.

## 1. Introduction

With the development of the economy and the improvement of life quality, People’s demand for meat has changed from quantity to quality. However, in recent years, the excessive pursuit of pig growth rate, feed conversion efficiency, and lean meat ratio has led to a decline in pork quality. In the production of pork, intramuscular fat (IMF) is attracting more and more attention. IMF directly affects the tenderness, flavor, and juiciness of pork [1]. The term IMF refers to the adipose tissue deposited between muscle fibers or bundles of muscle fibers, which is composed of triglyceride (TG), phospholipids, and cholesterol [2]. Fat droplets in muscle adipocytes and myoblasts are the main storage sites for TG. The results showed that the degradation of TG and phospholipids could produce aldehydes to improve pork flavor [3]. The deposition mechanism of IMF is a complex biological process. The factors affecting IMF deposition mainly include pig breed, age (slaughter weight), sex, nutrition, and environmental factors [4,5]. The nutrition strategy is a main method to improve pork quality and produce marbled pork. The Bama miniature pig is a local pig breed in Southern China, characterized by excellent reproductive performance, strong disease resistance, and tender meat. Research has shown that the Bama miniature pig has a newborn weight of about 450 g, reaches sexual maturity at 90–120 days of age, and weighs up to 40 kg as an adult pig [6]. Because of its small size, rapid growth and development, and easy feeding management, it is a principal experimental animal model.

Succinic acid is a common natural organic acid, that is produced by the microbial fermentation of cellulose in feed. Studies have shown that dietary cellulose increases the level of succinic acid in the caecum of mice, and this increase is even more pronounced when sodium succinate is added to a high-fat diet [7]. Succinic acid, one of the intermediate products of the tricarboxylic acid cycle, plays an important role in metabolism. Numerous reports in the past have only revealed that pig dietary 1% succinate supplementation regulates intestinal inflammation and hepatic bile acid metabolic homeostasis through the succinate-SUCNR1 axis [8,9]. Endogenous succinate has traditionally been regarded as a danger signal to promote an inflammatory response in the gut or the whole body [10,11]. However, other studies have also shown that a 1% succinate supplement in the diet does not cause diarrhea in pigs, but a concentration of more than 1% leading to excessive accumulation of succinate in the colon can cause diarrhea [12]. Some reports showed that succinic acid supplementation could improve the anti-stress ability and disease resistance of chickens and obviously promote lipase activity and insulin activity [13,14]. In addition, the glucose tolerance and insulin sensitivity of mice were improved by adding an appropriate amount of succinate to a high-fat diet [9]. Furthermore, succinic acid can be converted to glucose by gluconeogenesis, which provides energy to the body. For example, succinate promotes skeletal muscle protein deposition through the extracellular-signal-regulated kinase/protein kinase B signaling pathway [15]. These studies suggest that succinate, as an important fermentation product of gut microbes and an important intermediate metabolite of the tricarboxylic acid cycle, may regulate glucose and lipid metabolism in the body. However, the role of exogenous succinic acid supplements in lipid metabolism remains largely unknown.

On the other hand, the increase in succinate concentration will lead to an increase in the level of succinylation modification of proteins in the body. Succinylation is a specific type of protein modification mediated by succinyl coenzyme A, in which the succinyl group binds to the lysine site of the protein. Of mitochondrial proteins, 30% are succinylated [16]. In contrast to acetylation, succinylated modification promotes aerobic oxidation of glucose in glucose metabolism [17]. Sirtuin5 (SIRT5) is the most reported enzyme that regulates succinylation, and its function is to decrease protein succinylation [18]. Studies have shown that succinylation in adipose tissue regulates three important proteins: Glutamate dehydrogenase, succinate dehydrogenase, and mitochondrial uncoupling protein 1 [19]. SIRT5 knockout of brown adipose tissue in mice leads to hyper-succinylation of these proteins, which in turn leads to mitochondrial respiratory impairment, defective mitophagy, and lipid metabolic disorder [19]. Recent reports have revealed that dietary disodium succinate regulates lipid synthesis, gluconeogenesis, and glycogen synthesis in intestinal, liver, and muscle tissues, thereby inhibiting protein degradation in tissues, increasing fat deposition, and improving glucose tolerance [20]. Mechanistically, it works by regulating the levels of succinylation of the tricarboxylic acid cycle and fatty acid degradation-related proteins. However, it has not been reported whether succinate can regulate glucose and lipid metabolism and then affect fat deposition and meat quality in pigs.

In this study, a Bama miniature pig was used as a model to investigate the effects of dietary succinate supplementation on growth performance, carcass traits, meat quality, and plasma metabolites. We further revealed the mechanism of the regulation of porcine lipid synthesis and metabolism by sequencing the longissimus dorsi muscle (LM) metabolome and culturing porcine preadipocytes in vitro.

## 2. Materials and Methods

### 2.1. Animals, Diets, and Treatments

This experiment was conducted on a commercial pig farm in Henan province, China. All animal care and experimental procedures were approved in advance by the Animal Care and Use Committee of Northwest A&F University (TNWSUAF, 9 May 2020) and followed Chinese animal welfare guidelines. Twelve 28-day-old Bama mini-pigs were randomly divided into two groups (6 replicates per group), the control group (CON), and the sodium succinate group (SUC). The CON group was fed a basal diet consisting of a corn–soybean meal, while the SUC group was fed a basal diet supplemented with 1% sodium succinate (Aladdin, Shanghai, China). The basal diet was formulated according to the Chinese National Feeding Standard for Swine (Ministry of Agriculture of the People’s Republic of China, 2004). The composition and nutrient levels of the basal diet are shown in Table 1. The experimental period lasted for 90 days. All pigs were provided free access to feed and water throughout the experiment. Body weight and food intake were measured at 30-day intervals.

### 2.2. Cell Culture and Treatments

The primary preadipocytes were isolated from the adipose tissue of 3-day-old piglets by the method of primary digestive cells. The cells were cultured in DMEM/F12 (10565-018, Gibco, CA, USA) medium containing 15% fetal bovine serum (FBS) (10099-141, Gibco, CA, USA), 10,000 units/mL penicillin, and 10,000 μg/mL streptomycin (15140-122, Gibco, CA, USA). After 60% confluence, the cells were cultured for 24 h in growth medium supplemented with 0, 5, 10, 20 mM dimethyl succinate (DMS) (Abmole, M10425, Houston, TX, USA). Then the adipogenic differentiation of porcine primary preadipocytes was performed. Adipocyte differentiation was induced for 48 h in an induction medium containing 10% FBS, 0.5 mM isobutyl methylxanthine (Sigma, St. Louis, MO, USA), 0.25 μm dexamethasone (Sigma), 1 μg/mL insulin (Sigma), and 1 μm rosiglitazone (Sigma). After two days, the cells were replaced with medium containing 10% FBS and 1 μg/mL insulin. The cells were collected 7 days after differentiation.

### 2.3. Sample Collection

After 90 days of sodium succinate treatment, all pigs were fasted for 12 h and then euthanized using electric shock (120 V, 200 Hz) and bloodletting. Blood was then collected and centrifuged at 4 °C and 3500× *g* for 10 min to obtain serum. After bleeding and shedding, the pig’s head, hooves, tail, and innards (with suet and kidneys retained) were removed to preserve the carcass. Hot carcass weight was recorded individually and calculated dressing percentage. Back fat depth was calculated by averaging the first, last rib, and last lumbar vertebrae values. The LM area was measured at the 10th rib. The carcass was divided into anterior, middle, and posterior parts, which were stripped of bone, muscle, skin, and fat, respectively. When the carcass is stripped, intermuscular fat counts as muscle and is not removed; lean meat on bones should be stripped clean. The operating loss in the stripping process is not more than 2%. Bone, fat, and muscle were weighed and divided by carcass weight to calculate bone, fat, and muscle rates. A total of 400 g of LM meat was collected within 20 min after slaughter and stored at 4 °C for meat quality evaluation. The LM, subcutaneous adipose tissue (SAT), IMF, and visceral adipose tissue (VAT) were collected from each pig and put into liquid nitrogen for qPCR, Western blotting, and metabolome analysis. The LM, SAT, and liver were formalinized for histological analysis.

### 2.4. Meat Quality Analysis

A pH meter (DK-2730, SFK-Technology, Copenhagen, Denmark) was used to test initial pH (pH45min) and final pH (pH24h) values at 45 min and 24 h after slaughter. Fresh cross-sections of LM were cut 3 h after slaughter and placed on a white porcelain plate. The flesh color was evaluated by the visual scoring method under normal indoor light. The LM samples at the same position were stored at 4 °C for 24 h. The fresh cross-sections were cut with a knife, and the marbling was measured according to the marbling score card. The Marbling scorecard and Flesh Color scorecard refer to the U.S. official color and marbling standards (NPPC). The drip loss was determined following the procedure as described by Honikel [21]. The cooking yield and shear force (N) were determined according to the previously described methods [22]. The LM meat (about 50 g) was cut into 2–3 mm thin slices and placed in a vacuum freeze-dryer (model 4.5, Labconco Corp, Fort Scott, KS, USA) for freeze-drying for 72 h, then milled into powder. The intramuscular fat content was determined by the Soxhlet extraction method.

### 2.5. Serum Biochemical Indices

Glucose, insulin, malondialdehyde (MDA), TGs, total cholesterol, high-density lipoprotein (HDL-C), low-density lipoprotein (LDL-C), and free fatty acids (FFA) were measured in serum using an automated microplate reader (The Beckman Company, Brea, CA, USA) and a commercial kit (Beyotime, Shanghai, China) according to the instructions.

### 2.6. Histological Analysis

LM and SAT were fixed in a 4% paraformaldehyde solution overnight, then paraffin-embedded and cut to 5 μm thick. Samples were stained with hematoxylin and eosin (H&E) to assess morphological changes in the tissues. In addition, livers from three pigs per group were selected for embedding at the optimal cutting temperature. After the samples were cut to a thickness of 8 μm, they were stained with oil red O to observe the accumulation of lipid droplets in the liver. Finally, the stained images were obtained by light microscopy.

### 2.7. Succinate Determination

Succinic acid was determined with a succinic acid colorimetric assay kit (Sigma-aldrich, St. Louis, MO, USA) according to the method of a previous study [8]. Briefly, a standard succinate solution of 0, 2, 4, 6, 8, 10 nmol/Well was first prepared in a 96-well plate. Then 10 mg of tissue or 200 μL of serum were put into the precooled succinate assay buffer, quickly homogenized, and the supernatant was obtained by centrifugation. Then 2 μL of sample solution was added to each well in a 96-well plate, and the configured reaction mixture was added to make up 50 μL. The 96-well plate was set at 37 °C for 30 min, and the absorbance was measured at 450 nm. The concentration of each sample was calculated by absorbance.

### 2.8. BODIPY Dyeing

Bodipy dye solution from Beyotime (Shanghai, China). Porcine preadipocytes treated with dimethyl succinate were induced to differentiate and fixed with 4% paraformaldehyde for 30 min. It was then stained with a bodipy staining solution for 30 min, followed by dapi staining for 5 min. The results were observed using Fluorescence microscope (Biotek, Winooski, VT, USA).

### 2.9. Metabolomics Analysis

Firstly, all pig LM samples were extracted by the organic reagent precipitation method, and quality control (QC) samples were prepared at the same time. The samples were randomly sorted and tested, and QC samples were inserted before, during, and after the samples as a repeated evaluation of experimental techniques. The samples were detected by mass spectrometry in positive and negative ion modes. The secondary spectra were generated by using fragment information and were matched with the standard secondary spectra in the public database. The matching results were scored, and the secondary identification results of metabolites were obtained. XCMS software was used to extract the signal intensity information of each substance in different samples, and metaX software was used for quality control. First, low-quality peaks were removed (more than 50% missing in QC samples or more than 80% missing in actual samples), the missing values were then filled with Knn (K-nearest Neighbors), followed by Probabilistic Quotient Normalization and QC-robust spline batch correction. The detection intensity information of each metabolite in different samples was displayed by the HEATMAP map. In this study, fold-change and independent-sample *t*-tests were used for statistical analysis. The q-value was obtained by BH correction, and the variable important for the projection value was obtained by PLS-DA combined with multivariate statistical analysis, screening for differentially expressed metabolic ions. Differential metabolites were shown using volcano plots (Univariate Statistical Test). The enrichment of the kyoto encyclopedia of genes and genomes (KEGG) was analyzed by GGPLOT2. The results were shown by a scatter plot.

### 2.10. Quantitative Real-Time PCR Analysis

Total RNA was extracted from tissues and cells using TRIzol (Takara, Tokyo, Japan), followed by reverse transcription using the PrimeScript RT kit (Takara). The resultant complementary DNA was subjected to quantitative real-time PCR using SYBR Premix Ex Taqs (Takara). The procedure for quantitative real-time PCR was as follows: incubation at 95 °C for 10 min, denaturation at 95 °C for 15 s (with 40 cycles), annealing at 60 °C for 60 s, and extension at 72 °C for 30 s. Gene expression levels in each sample were calculated relative to β actin mRNA levels according to the 2^−ΔΔCT^ method. All primers are shown in Table 2.

### 2.11. Immunoblotting Analyses

Immunoblotting was performed according to methods previously studied in our laboratory [23]. Protein was first extracted from adipose tissue and cells using lysis buffer (Solarbio, Beijing, China). Approximately 30 μg of protein was separated by electrophoresis (12% and 15% SDS-PAGE gels) and then transferred to PVDF nitrocellulose membranes (Millipore, MA, USA), which were then blocked by 5% skim milk for 2 h. After blocking, these membranes were incubated overnight with various primary antibodies, including anti-PPARG, anti-FABP4, anti-c/EBP α, anti-ATGL, anti-Succinyllysine, anti-Acetyl Lysine, and anti-β-Actin (ABCAM, Cambridge, UK). The membranes were then washed and incubated with HRP-conjugated secondary antibodies for 2 h. Proteins were imaged using chemiluminescent Peroxidase substrates (Millipore) and quantified using a ChemiDoc XRS system (Bio-Rad, Richmond, CA, USA).

### 2.12. Statistical Analysis

In this experiment, all data were tested for normal distribution using SPSS 20.0 software (IBM Corp., Armonk, NY, USA). Then the student’s *t*-test was used to analyze the significant difference between the two groups. Data are presented as mean ± SD. *p* < 0.01 ** was considered extremely significant, and *p* < 0.05 * was considered significant.

## 3. Results

### 3.1. Sodium Succinate Improves the Growth and Meat Quality of Pigs

As shown in Table 3, there was no significant effect of sodium succinate supplementation on average daily feed intake in pigs. With succinate supplementation, the weight of 30-and 60-day-old pigs did not change significantly, but at 90 and 120-day-old pigs, the weight of the SUC group was significantly higher than that of the CON group (*p* < 0.05). Consistently, at 61–120 days of age, the average daily gain in the SUC group was significantly higher than that in the CON group (*p* < 0.05). At the age of 91–120 days, the feed/gain ratio of the SUC group was lower than that of the control group (*p* < 0.05).

The effects of dietary sodium succinate supplementation on carcass traits are shown in Table 4. The backfat depth, eye muscle area, and total fat in the SUC group were significantly higher than those in the CON group (*p* < 0.05). Sodium succinate supplementation had no effect on carcass weight, muscle, or bone content. The effect of dietary sodium succinate supplementation on meat quality is shown in Table 5. Compared with the CON group, the shear force in the SUC group was significantly lower (*p* < 0.05). At the same time, 1% sodium succinate supplementation increased the marbling score and intramuscular fat content of LM (*p* < 0.05). However, no significant differences were observed in pH, meat color score, water-holding capacity, or cooking loss. These data suggest that dietary sodium succinate supplementation improves meat quality in pigs.

### 3.2. Sodium Succinate Promotes Lipid Accumulation in Pig Serum, Adipose Tissue, and Liver

**Figure 1:**
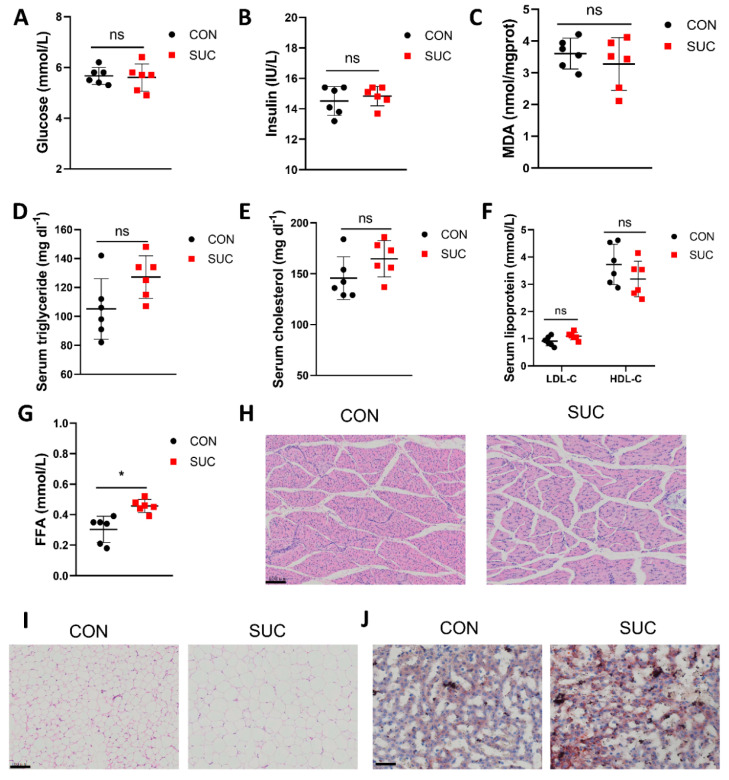
Effects of succinate supplementation on serum biochemistry, muscle development, fat, and liver lipid deposition in pigs. (**A**–**G**) Effects of dietary succinate supplementation on serum glucose, insulin, MDA, triglycerides, cholesterol, lipoproteins, and FFA. (**H**) H & E staining was used to detect the effect of succinate supplementation on LM muscle fiber. Scale bar: 100 μm. (**I**) H & E staining was used to detect the effect of succinate supplementation on lipid droplets in subcutaneous adipose tissue. Scale bar: 100 μm. (**J**) The effect of dietary succinate supplementation on hepatic lipid droplets was detected by oil red O staining. Scale bar: 100 μm. * *p* < 0.05; ns: not significantly different.

We further investigated the effects of sodium succinate on serum lipids, glucose metabolism, and antioxidants. The results showed that dietary sodium succinate had no effect on serum glucose, insulin, MDA, TG, cholesterol, LDL-C, and HDL-C levels (Figure 1A–F). But compared with the CON group, the SUC group had significantly higher FFA levels (Figure 1G, *p* < 0.05). Dietary sodium succinate decreased the cross-sectional area of LM (Figure 1H). Of note, the SAT adipocytes in the SUC group were significantly larger and had more hepatic fat deposits (Figure 1I,J). These results indicate that sodium succinate improves meat quality by increasing lipid deposition in the adipose tissue and liver of pigs.

### 3.3. Sodium Succinate Regulates the Lipid Composition of LM

**Figure 2:**
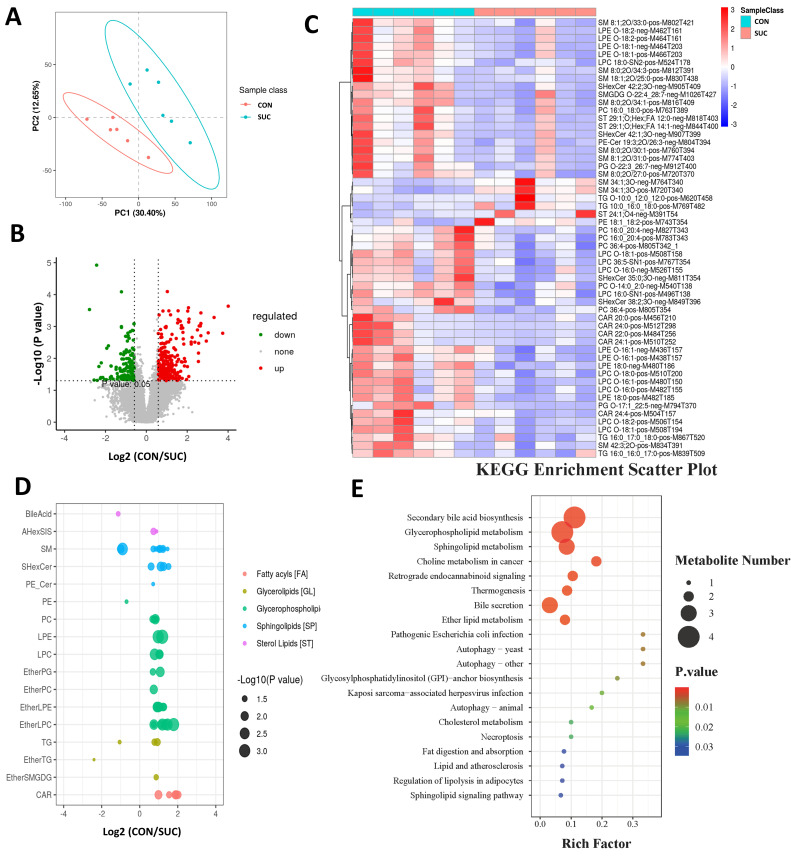
Effects of dietary succinate supplementation on LM metabolomics. (**A**) The Plsda model was used to show the classification ability between the two groups. (**B**) Volcano map of different metabolites between the two groups. (**C**) Heat map of key metabolites between the two groups. (**D**) Effects of dietary succinate supplementation on the lipid composition of LM. (**E**) KEGG enrichment analysis of different metabolites between the two groups.

To further resolve the effect of sodium succinate on fat deposition in porcine muscle, we performed a non-targeted metabolomic analysis of the LM. As shown in Figure 2A, the PLS-DA models showed good classification ability, indicating that differential metabolite analysis was accurate. Compared with the SUC group, 272 metabolites were significantly up-regulated and 156 metabolites were significantly down-regulated in the CON group (Figure 2B). The heat map shows some of the differential metabolites (Figure 2C). We further found that sodium succinate mainly affected the lipid metabolism of LM, including fatty acids, TG, glycerophospholipids, sphingolipids, and so on (Figure 2D). Finally, KEGG enrichment analysis revealed that sodium succinate, in addition to affecting secondary bile acid synthesis, also regulates signaling pathways such as lipid synthesis and lipolysis, autophagy, and thermogenesis (Figure 2E).

### 3.4. Succinate Regulates the Expression of Genes Related to Lipid Metabolism

**Figure 3:**
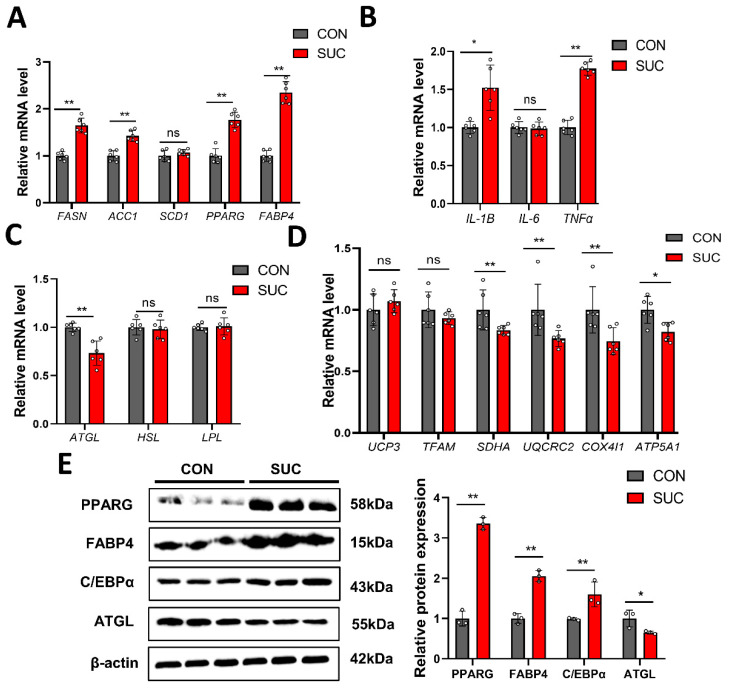
Effects of dietary succinate supplementation on the expression of IMF key genes. (**A**) Effects of dietary succinate supplementation on the expression of IMF lipid synthesis genes. (**B**) Effects of dietary succinate supplementation on the expression of IMF inflammatory genes. (**C**) Effects of dietary succinate supplementation on the expression of IMF lipid metabolism genes. (**D**) Effects of dietary succinate supplementation on the expression of IMF energy metabolism genes. (**E**) Immunoblotting was used to detect the protein level of related differential genes. * *p* < 0.05; ** *p* < 0.01; ns: not significantly different.

**Figure 4:**
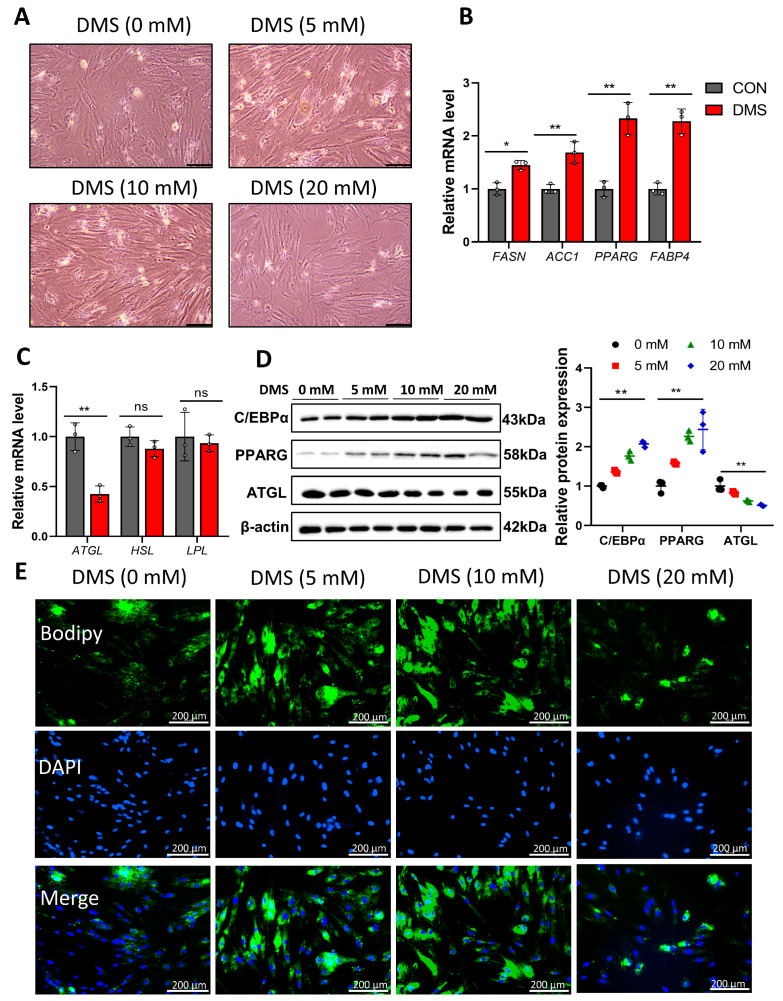
Effect of succinate on adipogenic differentiation of porcine primary preadipocytes. (**A**) The primary porcine preadipocytes were treated with DMS for 24 h to induce their adipogenic differentiation. Scale bar: 100 μm. (**B**,**C**) qPCR was used to detect the expression of genes related to lipid synthesis and lipid metabolism. (**D**) The differential gene protein expression was detected by Western blotting. (**E**) Bodipy was used to detect lipid droplets. Scale bar: 200 μm. * *p* < 0.05; ** *p* < 0.01; ns: not significantly different.

Considering that sodium succinate regulates LM lipid synthesis and catabolism, we further examined the mRNA and protein levels of some key genes in the IMF. Compared with the CON group, the SUC group had significantly up-regulated expression of fatty acid synthesis enzyme (FASN), acetyl-cocarboxylase 1 (*ACC1*), peroxisome proliferator-activated receptor gamma (*PPARG*), fatty acid-binding protein 4 (*FABP4*), and genes related to inflammation (*IL1B* and *TNF-α*), while down-regulating genes related to lipolysis (*ATGL*) and energy metabolism (*SDHA*, *UQCRC2*, *COX4I1,* and *ATP5A1*) (Figure 3A–D). The results of Western blotting were consistent with qPCR (Figure 3E and Figure S1). We further isolated and cultured pig primary preadipocytes and established an ectopic metabolite treatment (cell-permeable DMS) strategy to assess the adipogenic differentiation ability of the cells (Figure 4A). qPCR and Western blot results showed that DMS significantly promoted the expression of genes related to lipid synthesis and decreased the level of ATGL in porcine primary preadipocytes (Figure 4B–D and Figure S2). Further BODIPY staining showed that DMS promoted the accumulation of lipid droplets in porcine primary preadipocytes, with the best effect at a concentration of 10 mM (Figure 4E). These data suggest that succinate promotes adipogenic differentiation of porcine primary adipocytes through regulation of lipid metabolism-related genes.

### 3.5. Succinate Promotes Lipid Deposition by Regulating Succinylation

**Figure 5:**
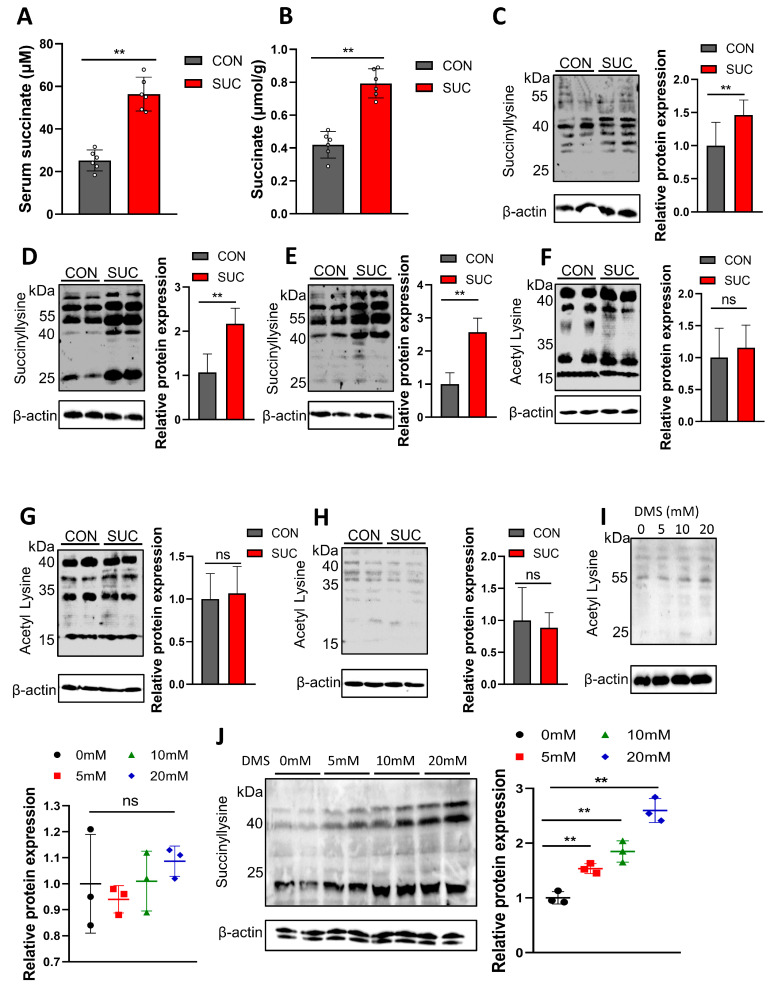
Effects of succinate on acetylation and succinylation in porcine adipose tissue. (**A**,**B**) Levels of succinate in serum and adipose tissue. (**C**) Effects of dietary succinate supplementation on succinylation of adipose tissue in pigs. (**D**) Effects of dietary succinate supplementation on succinylation of intermuscular adipose tissue in pigs. (**E**) Effects of dietary succinate supplementation on succinylation of subcutaneous adipose tissue in pigs. (**F**–**H**) Effects of succinate supplementation on the acetylation of visceral fat, intermuscular fat, and subcutaneous fat in pigs. (**I**) Effect of DMS on acetylation of porcine primary preadipocytes. (**J**) Effect of DMS on succinylation of porcine primary preadipocytes. ** *p* < 0.01; ns: not significantly different.

To further understand the mechanism by which succinate promotes lipid deposition, we examined the level of succinylation. As shown in Figure 5A,B, sodium succinate treatment significantly increased succinic acid levels in serum and adipose tissue. Consistently, SUC significantly increased the succinylation of VAT, IMF, and SAT compared with controls (Figure 5C–E and Figure S3). However, there was no significant change in acetylation modification between the groups (Figure 5F–H and Figure S4). Consistent with in vivo data, DMS increased cellular succinylation levels but had no effect on acetylation in porcine primary preadipocytes (Figure 5I,J and Figure S5). Thus, succinate promotes pig fat deposition by increasing levels of succinylation rather than acetylation.

## 4. Discussion

Several studies have shown that succinate, a tricarboxylic acid cycle intermediate, can affect a variety of metabolic processes, such as redox state, phosphate metabolism, carbon utilization, amino acid incorporation, and fatty acid synthesis [24,25,26]. For example, the addition of 0.05% succinic acid to cultures of acetobacter pasteurii increased intracellular ATP content by 185%. This is because the addition of succinic acid may promote oxidative dehydrogenation of succinic acid to fumaric acid; it releases more reduced nicotinamide adenine dinucleotide, which passes through the respiratory chain and eventually makes more ATP [9,27]. Therefore, we suggest that adding sodium succinate to pig feed may affect nutrient metabolism through the tricarboxylic acid cycle and thus promote pig growth (energy deposition). Our results showed that 1% succinate significantly increased daily gain and feed/gain ratio but had no effect on feed intake. Furthermore, we found that the addition of succinate promoted pig fat deposition, especially subcutaneous fat and intramuscular fat content. Consistent with our results, the addition of 0.15% succinate to the zebrafish diet promoted the increase in crude protein, crude fat, and energy and significantly altered the growth and development of zebrafish [20]. A study of succinate in rats found that feeding sodium succinate increased energy expenditure but did not affect dietary intake and that succinate could enter the body as a substrate [28]. Li et al. reported that 1% succinate supplementation inhibited daily the gain and feed intake of piglets [8]. This is contrary to our results. The difference may be due to the fact that succinate treatment did not lead to a change in pig intake in our results. In addition, fat deposition capacity (marbling formation) will increase with age. Lee et al.’s study found succinate supplementation at 28–56 days of age, when fat deposition capacity is weak, so the effect of succinate is not prominent. Our results also showed no significant change in body weight at 30–60 days of age, whereas the critical period for succinate-induced weight gain was 90–120 days of age (after sexual maturation) in Bama miniature pigs. In conclusion, succinate promotes weight gain, which is mainly related to lipid deposition.

The key factors affecting meat quality are color, flavor, tenderness, juiciness, and water holding capacity. The study found that tenderness, juiciness, and flavor also increased significantly when the IMF content was increased to 2.5% [29]. The results showed that increasing dietary succinate levels increased IMF content and decreased shear stress, but the cooking and dripping losses did not change. This suggests that succinate mainly affects muscle fat deposition and tenderness but has no effect on muscle water holding capacity. In addition to IMF content, the type and structure of muscle fibers are the key factors determining muscle tenderness. A series of studies have demonstrated that succinate induces the transition of skeletal muscle fiber via the SUNCR1 signaling pathway [30,31]. This is consistent with the reduction in LM cross-sectional area by succinate in this study. This further explains the decrease in shear stress in this study, which is related to the change in muscle fiber type caused by succinate. Unlike studies on the regulation of muscle fiber function, the effects of succinate on lipid metabolism are rarely studied. Therefore, this study focused on the effect of succinate on fat deposition in pigs. Our results indicate that succinate supplementation increases serum fatty acid levels and increases lipid accumulation in adipose tissue, liver, and LM. These results indicate that succinate does promote fat deposition. Succinate promoted adipogenic differentiation of porcine preadipocytes in vitro. Numerous studies have demonstrated that succinate is involved in the tricarboxylic acid cycle as a substrate and is converted to pyruvate, which is mainly involved in liver fatty acid synthesis and gluconeogenesis [24,32,33]. Excess glucose and fatty acids in the liver are converted into fat for storage or transferred to muscle and adipose tissue for storage. It can be concluded that the addition of 1% succinate to the diet fed to pigs resulted in an increase in fat accumulation and glycogen synthesis in the liver and muscle of pigs, which in turn improved meat quality and brought greater benefits to farming production.

The metabolome results of LM in this study showed that succinate significantly altered the composition of lipids in muscle, particularly fatty acids, triglycerides, glycerophospholipids, and sphingolipids, compared with the control group. Further qPCR analysis showed that succinate significantly up-regulated the expression of FASN, ACC1, FABP4, CCAAT/enhancer binding proteins alpha (C/EBP α), and PPARG, while decreasing the expression of ATGL. FASN and ACC1 are the key rate-limiting enzymes of fatty acid synthesis [34]. Most of the fatty acids required for body fat deposition come from de novo fatty acid synthesis. Therefore, these two genes are often important candidate genes for body fat deposition and meat quality traits. Transcription of PPARG is essential for adipocyte differentiation, whereas it mainly induces some adipocyte-specific gene activation at the end of adipocyte differentiation [35,36]. Both promote the accumulation of lipids in fat cells. Our results also show that succinate does promote the differentiation of porcine preadipocytes and induce more lipid droplets.

Succinylation is a new posttranslational modification, but its biological function is very powerful. Although the discovery of succinylation is still in progress, some important biological functions have been reported, such as energy metabolism, cancer progression, and the browning of white fat [37]. Succinylation is widespread in mitochondria. Brown adipose tissue is rich in mitochondria and is a key tissue for heat production and energy expenditure. The widespread presence of succinylated modifications underscores their importance in the development of obesity. For example, succinate supplementation promotes fetal brown fat development, which is associated primarily with enhanced succinylation modification of white fat browning-related proteins, thereby protecting offspring mice from diet-induced obesity [38]. Similarly, brown adipose tissue-specific deletion of SIRT5 leads to a dramatic increase in global protein succinylation and acrylamide. This leads to reduced function of thermogenic and other proteins in brown fat, triggering mitochondrial respiratory impairment, defective mitophagy, and metabolic inflexibility [19]. Although there are no relevant regulatory genes for white fat brown in pigs, our results show that succinate significantly increases levels of succinylation in the adipose tissue pool, which in turn promotes fat deposition. At present, we have not identified the main target protein of succinylation modified by succinate, but this provides a direction for future research.

## 5. Conclusions

Adding 1% succinate to the diet increased growth performance, backfat depth, and intramuscular fat content in Bama miniature pigs, affecting levels of fatty acids, triglycerides, glycerophospholipids, and sphingolipids in LM and improving the quality of meat. Further analysis revealed that succinate promotes adipogenic differentiation of porcine primary adipocytes by upregulating levels of succinylation modifications that affect the expression of lipid synthesis and lipid breakdown genes. This study provides data support and a theoretical basis for the effective application of succinate in the pig industry.

## Figures and Tables

**Table 1:** Composition and nutrient levels of the basal diet.

Ingredients	Percentage (%)	Nutrient Levels	Content
Corn	65.76	DE, Mcal/kg	3.40
Soybean meal	18.60	CP, %	16.65
Soybean oil	2		
Whey powder	8		
Fish meal	2		
Lysine	0.43		
Methionine	0.08		
Threonine	0.13		
Tryptophan	0.02		
CaHPO_4_	1.05		
Calcium carbonate	0.68		
Salt	0.25		
Premix ^a^	1		
Total	100		

^a^ Vitamin mineral premix supplied the following per kilogram of diet: 4000 IU vitamin A (retinyl acetate), 400 IU vitamin D3 (cholecalciferol), 18 mg/kg vitamin E (α-tocopheryl acetate), 0.5 mg/kg vitamin K (menadione sodium bisulfate), 1.5 mg/kg vitamin B2 (riboflavin), 8 μg/kg vitamin B12 (cobalamin), 1.8 mg/kg pantothenic acid, 7.5 mg/kg nicotinic acid, 200 mg/kg Fe (from ferrous sulfate), 20 mg/kg Cu (from copper sulfate), 20 mg/kg Mn (from manganese sulfate), and 1.0 mg/kg I, and 0.2 mg/kg Se (sodium selenite).

**Table 2:** Primer sequences.

Genes	Primers	Sequences (5′ to 3′)
FASN	Forward	TGGGCATGGTGAACTGTCTC
	Reverse	GCGTGGTTGTTGGAAAGGTC
ACC1	Forward	GGCCATCAAGGACTTCAACC
	Reverse	ACGATGTAAGCGCCGAACTT
SCD1	Forward	TCTGGGCGTTTGCCTACTATCT
	Reverse	TCTTTGACGGCTGGGTGTTT
PPARG	Forward	AACATTTCACAAGAGGTGACCA
	Reverse	GATCTCGTGGACGCCATACT
FABP4	Forward	CAGGAAAGTCAAGAGCACCACAGGAAAGTCAAGA
	Reverse	TCGGGACAATACATCCTCCAACA
ATGL	Forward	ATGTTCCCCAAAGAGACGAC
	Reverse	GGCGAAGCGGGTTATGAT
HSL	Forward	CACAAGGGCTGCTTCTACGG
	Reverse	AAGCGGCCACTGGTGAAGAG
LPL	Forward	CGTGCTCAGATGCCCTACAA
	Reverse	AGACTCCACGTGCTGTTCCT
IL-1B	Forward	AAACCTTGACCTCAGCCCTC
	Reverse	CTCCTCCTTTGCCACAATCAC
IL-6	Forward	CTTCAGTCCAGTCGCCTTCT
	Reverse	CATCACCTTTGGCATCTTCTT
TNF-α	Forward	CCACCAACGTTTTCCTCACT
	Reverse	AATAAAGGGATGGACAGGGG
UCP3	Forward	TCACCTTCAGGACACGTTCG
	Reverse	AGGCATCCATCCTAGTGGGT
TFAM	Forward	TGCTTTGTCTACGGGTGCAA
	Reverse	GCAAAACTGAACGGAGAGCG
SDHA	Forward	ACATCAACGGAGGCAACACT
	Reverse	ACAGCCCATCCAGTTTCTCG
UQCRC2	Forward	CTCCTGTAAGGCGGTTGTGA
	Reverse	ACTGGATGCAAGACGAAGCA
COX4I1	Forward	GGTGGAGTCCCCTCTCGAT
	Reverse	GGATGGGGCCGTACACATAG
ATP5A1	Forward	TCGTGGTGTTCGTCTGACTG
	Reverse	TTTTCCCAACAGGGCTTGGT
β-Actin	Forward	AACGGCTCCGGCATGTGCAA
	Reverse	CTTCTGACCCATGCCCACCA

**Table 3:** Effects of dietary supplements with sodium succinate on the growth performance of the pigs.

Items	CON	SUC	*p*-Value
Body weight (kg)			
30 d	4.397 ± 0.170	4.398 ± 0.199	0.988
60 d	9.110 ± 0.201	9.227 ± 0.196	0.333
90 d	14.111 ± 0.249	14.555 ± 0.353	0.031
120 d	22.452 ± 0.641	24.097 ± 0.679	0.002
Average daily gain (kg/d)			
31–60 d	0.157 ± 0.001	0.161 ± 0.001	0.505
61–90 d	0.167 ± 0.008	0.178 ± 0.007	0.038
91–120 d	0.278 ± 0.026	0.318 ± 0.030	0.032
Average daily feed intake (kg/d)			
31–60 d	0.432 ± 0.021	0.435 ± 0.029	0.824
61–90 d	0.624 ± 0.016	0.630 ± 0.027	0.667
91–120 d	0.924 ± 0.039	0.935 ± 0.042	0.649
Feed/gain ratio			
31–60 d	2.763 ± 0.290	2.714 ± 0.277	0.772
61–90 d	3.750 ± 0.146	3.551 ± 0.186	0.066
91–120 d	3.339 ± 0.244	2.956 ± 0.257	0.024

Data are presented as mean ± SD. The replicates per group were 6.

**Table 4:** Effects of dietary supplements with sodium succinate on carcass traits of the pigs.

Items	CON	SUC	*p*-Value
Carcass weight (kg)	13.62 ± 0.55	13.88 ± 0.41	0.38
Dressing percentage (%)	65.70 ± 1.39	66.13 ± 0.90	0.54
Backfat depth (mm)	24.69 ± 0.66	26.33 ± 1.06	0.01
Eye muscle area (cm^2^)	7.17 ± 0.44	7.87 ± 0.26	0.01
Total muscle (%)	22.52 ± 1.14	22.24 ± 1.36	0.72
Total fat (%)	18.34 ± 0.73	20.13 ± 0.71	0.00
Total bone (%)	9.26 ± 0.37	8.95 ± 0.36	0.20

Data are presented as mean ± SD. The replicates per group were 6.

**Table 5:** Effects of dietary supplements with sodium succinate on the meat quality of the pigs.

Items	CON	SUC	*p*-Value
PH 45 min	6.27 ± 0.10	6.25 ± 0.10	0.79
PH 24 h	5.28 ± 0.12	5.27 ± 0.10	0.80
Meat color score	2.5 ± 0.55	2.33 ± 0.52	0.60
Drip loss (%)	3.55 ± 0.19	3.47 ± 0.19	0.46
Cooking yield (%)	65.35 ± 1.54	64.93 ± 1.27	0.61
Shear force (N)	56.03 ± 1.67	51.80 ± 2.05	0.00
Marbling score	1.33 ± 0.52	2.33 ± 0.52	0.01
Intramuscular fat content (%)	2.16 ± 0.24	2.86 ± 0.30	0.00

Data are presented as mean ± SD. The replicates per group were 6.

## Data Availability

The raw data supporting the conclusions of this article will be made available by the authors on request.

## Supplementary Materials

The following supporting information can be downloaded at: https://www.mdpi.com/article/10.3390/ani14070999/s1. Figure S1: Western blot membrane of PPARG (58 kDa), FABP4 (15 kDa), C/EBPα (43 kDa), ATGL (55 kDa) and β-actin (42 kDa) protein detected with anti-PPARG (1:1000; ABCAM, Cambridge, UK), anti-FABP4 (1:1000; ABCAM, Cambridge, UK), anti-C/EBPα (1:1000; ABCAM, Cambridge, UK), anti-ATGL (1:1000; ABCAM, Cambridge, UK) and anti-β-actin (1:1000; ABCAM, Cambridge, UK) antibodies, respectively; Figure S2: Western blot membrane of PPARG (58 kDa), C/EBPα (43 kDa), ATGL (55 kDa) and β-actin (42 kDa) protein detected with anti-PPARG (1:1000; ABCAM, Cambridge, UK), anti-C/EBPα (1:1000; ABCAM, Cambridge, UK), anti-ATGL (1:1000; ABCAM, Cambridge, UK) and anti-β-actin (1:1000; ABCAM, Cambridge, UK) antibodies, respectively; Figure S3: Western blot membrane of succinyllysine and β-actin (42 kDa) protein detected with anti-succinyllysine (1:1000; ABCAM, Cambridge, UK) and anti-β-actin (1:1000; ABCAM, Cambridge, UK) antibodies, respectively; Figure S4: Western blot membrane of acetyl Lysine and β-actin (42 kDa) protein detected with anti-acetyl Lysine (1:1000; ABCAM, Cambridge, UK) and anti-β-actin (1:1000; ABCAM, Cambridge, UK) antibodies, respectively; Figure S5: Western blot membrane of succinyllysine, acetyl Lysine, and β-actin (42 kDa) protein detected with anti-succinyllysine (1:1000; ABCAM, Cambridge, UK), anti-acetyl Lysine (1:1000; ABCAM, Cambridge, UK) and anti-β-actin (1:1000; ABCAM, Cambridge, UK) antibodies, respectively.
