# Supplementary material for: Effects of Succinate on Growth Performance, Meat Quality and Lipid Synthesis in Bama Miniature Pigs
Source: Animals (Basel). 2024 Mar 25;14(7):999. doi: 10.3390/ani14070999 (PMC11011074; doi:10.3390/ani14070999)
Supplement: Supplementary file 1 [file animals-14-00999-s001.zip › animals-2888765-supplementary.pdf]

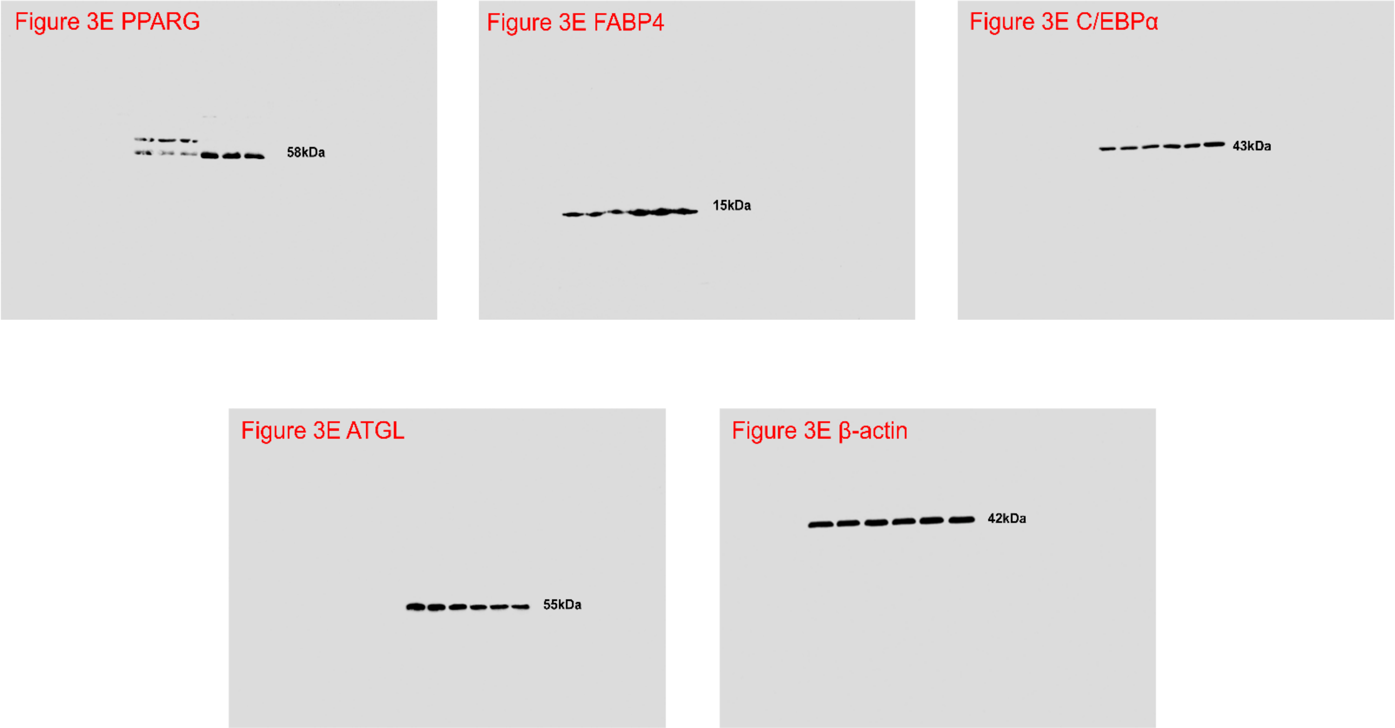

Figure S1: Western blot membrane of PPARG (58 kDa), FABP4 (15 kDa), C/EBPα (43 kDa), ATGL (55kDa) and β-actin (42kDa) protein detected with anti-PPARG (1:1000; ABCAM, Cambridge, UK), anti-FABP4 (1:1000; ABCAM, Cambridge, UK), anti- C/EBPα (1:1000; ABCAM, Cambridge, UK), anti- ATGL (1:1000; ABCAM, Cambridge, UK) and anti-β-actin (1:1000; ABCAM, Cambridge, UK) antibodies, respectively

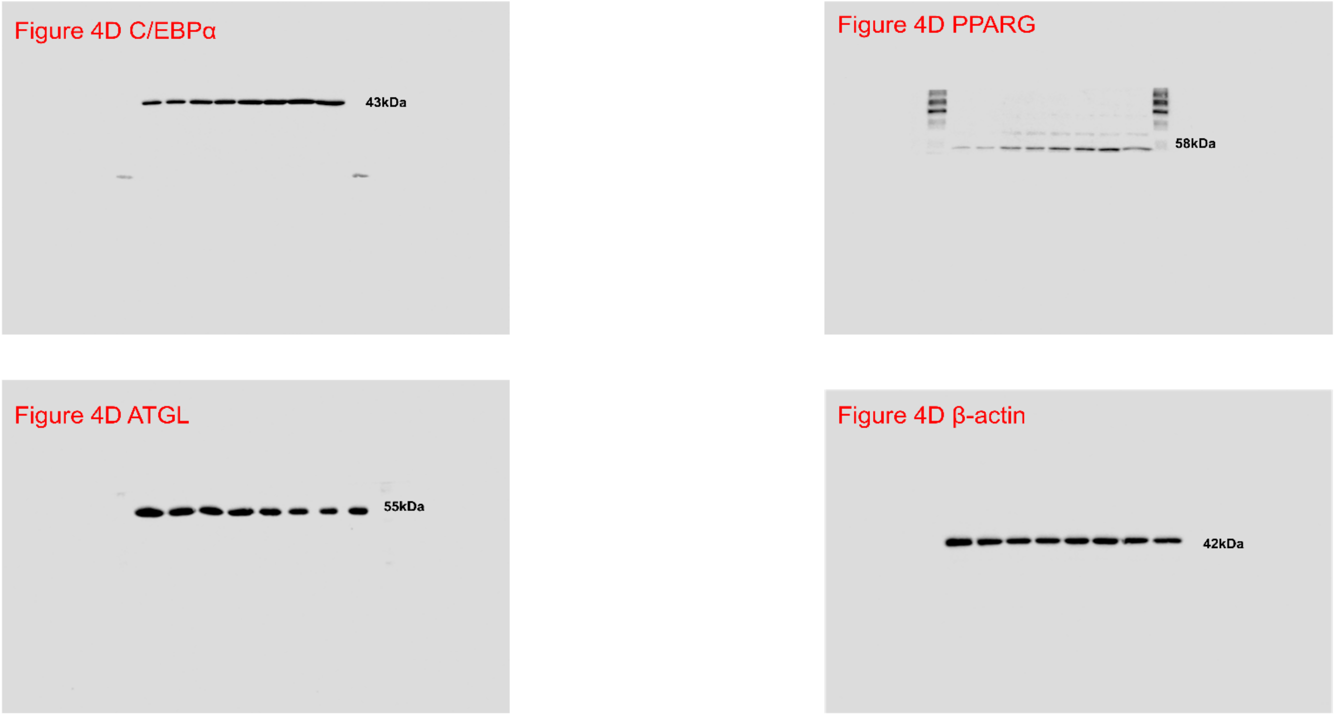

Figure S2: Western blot membrane of PPARG (58 kDa), C/EBPα (43 kDa), ATGL (55kDa) and β-actin (42kDa) protein detected with anti-PPARG (1:1000; ABCAM, Cambridge, UK), anti- C/EBPα (1:1000; ABCAM, Cambridge, UK), anti- ATGL (1:1000; ABCAM, Cambridge, UK) and anti-β-actin (1:1000; ABCAM, Cambridge, UK) antibodies, respectively.

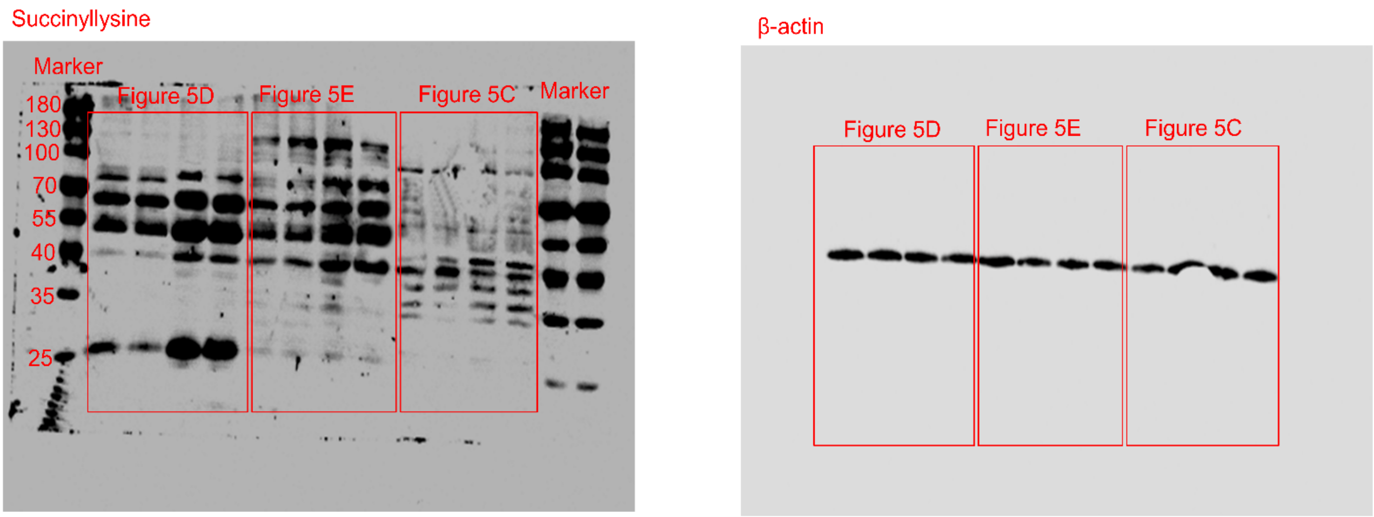

Figure S3: Western blot membrane of succinyllysine and β-actin (42kDa) protein detected with an-ti-succinyllysine (1:1000; ABCAM, Cambridge, UK) and anti-β-actin (1:1000; ABCAM, Cambridge, UK) antibodies, respectively.

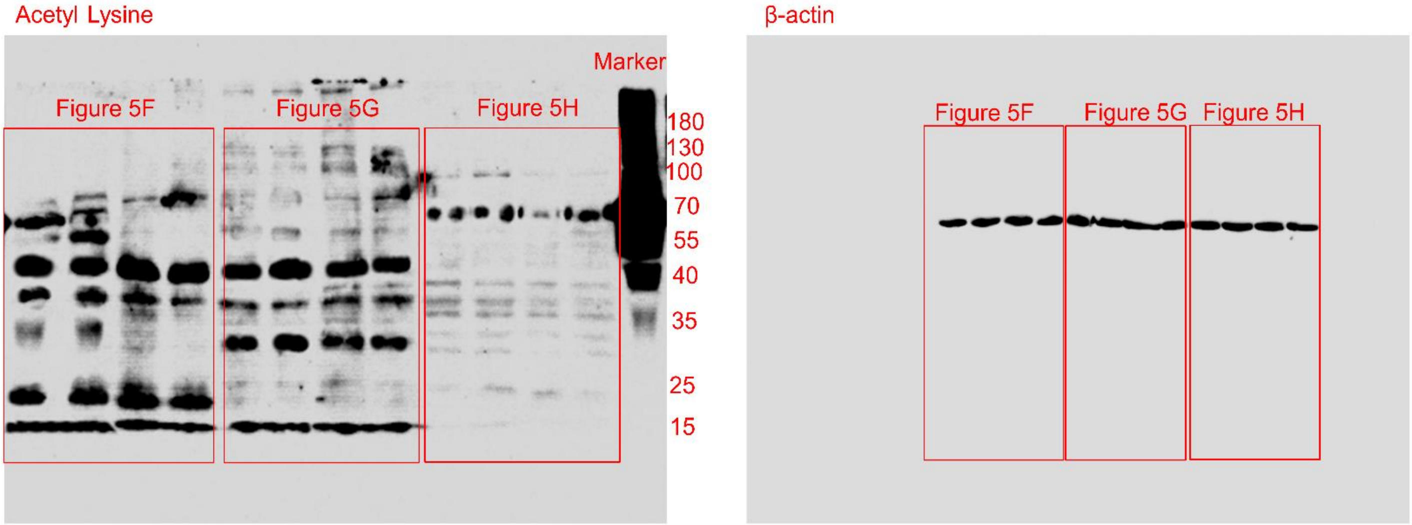

Figure S4: Western blot membrane of acetyl Lysine and β-actin (42kDa) protein detected with an-ti-acetyl Lysine (1:1000; ABCAM, Cambridge, UK) and anti-β-actin (1:1000; ABCAM, Cambridge, UK) antibodies, respectively.

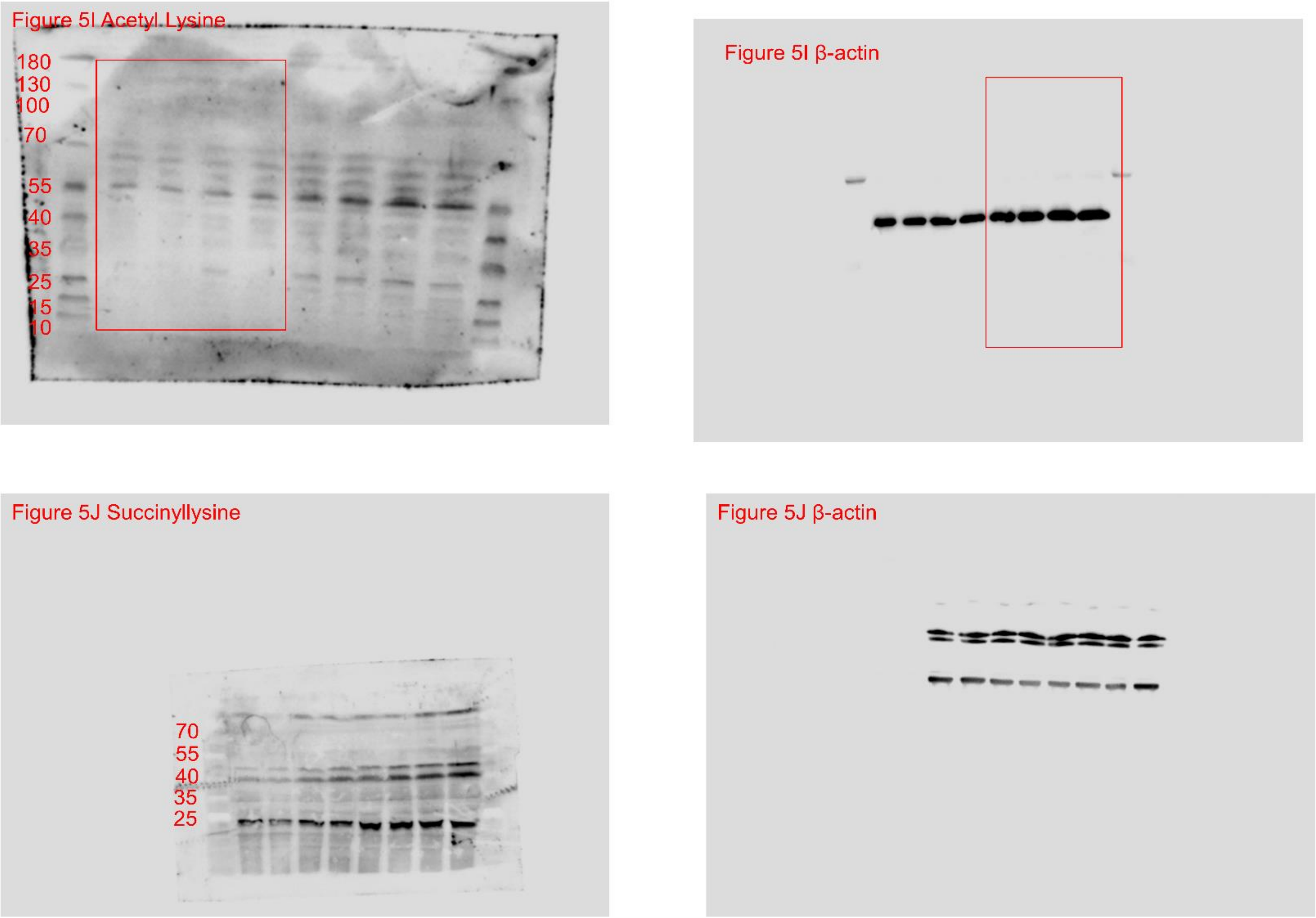

Figure S5: Western blot membrane of succinyllysine, acetyl Lysine and β-actin (42kDa) protein detected with anti-succinyllysine (1:1000; ABCAM, Cambridge, UK), anti-acetyl Lysine (1:1000; ABCAM, Cambridge, UK) and anti-β-actin (1:1000; ABCAM, Cambridge, UK) antibodies, respec-tively.
